# Best practice guidelines for the molecular genetic diagnosis of Type 1 (HFE-related) hereditary haemochromatosis

**DOI:** 10.1186/1471-2350-7-81

**Published:** 2006-11-29

**Authors:** Caitriona King, David E Barton

**Affiliations:** 1 National Centre for Medical Genetics, Our Lady's Children s Hospital, Crumlin, Dublin 12, Ireland

## Abstract

**Background:**

Hereditary haemochromatosis (HH) is a recessively-inherited disorder of iron over-absorption prevalent in Caucasian populations. Affected individuals for Type 1 HH are usually either homozygous for a cysteine to tyrosine amino acid substitution at position 282 (C282Y) of the *HFE *gene, or compound heterozygotes for C282Y and for a histidine to aspartic acid change at position 63 (H63D). Molecular genetic testing for these two mutations has become widespread in recent years. With diverse testing methods and reporting practices in use, there was a clear need for agreed guidelines for haemochromatosis genetic testing. The UK Clinical Molecular Genetics Society has elaborated a consensus process for the development of disease-specific best practice guidelines for genetic testing.

**Methods:**

A survey of current practice in the molecular diagnosis of haemochromatosis was conducted. Based on the results of this survey, draft guidelines were prepared using the template developed by UK Clinical Molecular Genetics Society. A workshop was held to develop the draft into a consensus document. The consensus document was then posted on the Clinical Molecular Genetics Society website for broader consultation and amendment.

**Results:**

Consensus or near-consensus was achieved on all points in the draft guidelines. The consensus and consultation processes worked well, and outstanding issues were documented in an appendix to the guidelines.

**Conclusion:**

An agreed set of best practice guidelines were developed for diagnostic, predictive and carrier testing for hereditary haemochromatosis and for reporting the results of such testing.

## Background

The term haemochromatosis was originally used by von Recklinghausen in 1889 [1] to describe tissue injury caused by increased levels of iron. A modern definition of haemochromatosis describes it as an inherited disorder of iron metabolism, characterized by inappropriately high absorption of iron by the gastrointestinal mucosa, leading to excessive storage of iron (particularly in the liver, skin, pancreas, heart, joints and testes) and ultimately resulting in impaired organ structure and function [2,3].

Haemochromatosis can be due to mutations in a number of genes (Table 1) but in terms of prevalence and phenotype, the most important type is that due to mutations in *HFE*, termed Type 1 hereditary haemochromatosis (HH). HFE-related HH arises predominantly in males between 40 and 60 years and it is particularly common in people of Northern European descent where it affects 1 in every 200–300 individuals. The condition has an autosomal recessive mode of inheritance and, depending on the population, 80–93% of clinically symptomatic individuals are homozygous for the HFE mutation, C282Y (G845A), with most of the remainder being compound heterozygotes for C282Y and H63D (C187G). Clinical expression is variable and a significant proportion of individuals with these genotypes do not develop the condition, which demonstrates low penetrance of the mutations and emphasises the need to define the genetic modifiers and environmental factors which contribute to iron overload in these individuals [4-7].

**Table 1:** Overview of genes involved in Hereditary Haemochromatosis (HH)

	**HFE-Related HH**	**Juvenile HH**		**TfR2-Related HH**	**Ferroportin-Related HH**
**OMIM Classification**	Type 1	Type 2, subtype A	Type 2, subtype B	Type 3	Type 4
**OMIM #**	235200	608374	606464	604720	606069
**Gene**	*HFE (formerly HLA-H)*	*HJV (formerly HFE2)*	*HAMP*	*TfR2*	*SLC4OA1*
**Gene Map Locus**	6p21.3	1q21	19q13.1	7q22	2q32
**Gene Product**	HFE	Haemojuvelin	Hepcidin	Transferrin Receptor 2	Ferroportin/Iron Regulatory Protein/Metal Transporter Protein
**Inheritance**	AR	AR	AR	AR	AD

AR: autosomal recessive; AD: autosomal dominant; OMIM: On-line Mendelian Inheritance in Man

Treatment of haemochromatosis usually involves removal of excess iron by weekly therapeutic phlebotomy (i.e., removal of blood) to reduce the serum ferritin concentration and the transferrin saturation. After initial treatment, normal iron levels are usually maintained by occasional phlebotomy. Specifics regarding treatment can be found in guidelines published by The British Committee for Standards in Haematology [8]. The earlier the diagnosis is made, and treatment to normalize serum iron studies commenced, the greater the likelihood that all the potential serious complications of HH can be prevented [3,5].

### Clinical presentation and reasons for referral

Early symptoms are relatively non-specific and include abdominal pain, weakness, lethargy and weight loss. Untreated individuals may develop hepatic fibrosis or cirrhosis and hepatocellular carcinoma develops in 25% of patients with established cirrhosis. In addition, untreated individuals may also develop progressive increase in skin pigmentation, diabetes mellitus, congestive heart failure and/or arrhythmias, arthritis and hypogonadism. Symptomatic individuals have biochemical evidence of iron overload (elevated serum transferrin saturation and ferritin concentration) and such biochemical evidence is found even in the absence of symptoms [2,4,5].

*Confirmatory diagnostic testing *currently involves molecular genetic testing for the C282Y and H63D mutations in the *HFE *gene or histological assessment of hepatic iron stores on liver biopsy. Most individuals with *HFE *related HH are either homozygotes for the C282Y mutation (80–93%) or compound heterozygotes for the mutations C282Y and H63D (<5%). Genetic testing for non-*HFE *related haemochromatosis is not widely available and diagnosis may have to be based on liver biopsy findings [4,5].

*Predictive testing *for at-risk relatives (e.g. siblings of genetically confirmed HH individuals) may be requested as well as *carrier testing *for family members.

*Pre-natal diagnosis *is not usually offered as the condition is a treatable, adult onset condition.

*Population screening *is not currently recommended primarily due to the penetrance issue surrounding the C282Y mutation [2].

## Methods

A survey of current practice in the molecular diagnosis of haemochromatosis was conducted by means of standard questionnaire. Based on the results of this survey, draft guidelines were prepared using the template developed by UK Clinical Molecular Genetics Society. A workshop was held to develop the draft into a consensus document. The consensus document was then posted on the Clinical Molecular Genetics Society website for broader consultation and amendment.

## Results

### The guidelines

#### Criteria for testing

The clinical criteria required for molecular genetic testing to proceed depend on local guidelines. Suggested biochemical criteria include elevated, fasting, serum transferrin saturation and persistently raised serum ferritin concentration. Numerically, local biochemical testing methodologies will dictate what constitutes elevated transferrin saturation but a result of 45% would generally be accepted for genetic testing, particularly to facilitate early detection of iron overload. A value exceeding the local upper limit of normal constitutes an elevated concentration of ferritin. However, an elevated ferritin is not specific for HH and therefore should be considered in conjunction with indicators of inflammation and liver disease.

Iron studies are not necessary for predictive testing but such cases may benefit from a referral to a clinical genetics service.

Carrier testing is routinely offered to close relatives of confirmed cases or of confirmed carriers. When devising a carrier testing policy, it is worth considering whether more distant relatives have carrier risks elevated above that of the general population.

#### Testing strategy

A large number of methods are used by laboratories. Most use a combination of PCR and restriction enzyme digest for one or both mutations. Other methods employed include real time PCR (LightCycler), allele-specific PCR, heteroduplex analysis and PCR-SSCP. The EMQN and UKNEQAS assessors have commented on the small number of mistakes and general reliability of the wide range of different technological approaches used.

It is important to note that the S65C variant (A193T), which is just six bases away from the H63D mutation, has been reported to interfere with H63D analysis for some methods such as the LightCycler [9] and the Stott duplex method [10] (Mark Hill, Kieran Guinan; personal communications), such that H63D/S65C compound heterozygotes appear as H63D homozygotes. Therefore, apparent H63D homozygotes detected by such methods must be subjected to further analysis to out rule interference by S65C giving an erroneous result.

The method in the original paper describing the *HFE *gene may be compromised by a sequence variation under the reverse primer for C282Y, under some conditions [11] and a modified primer [12] should be used instead. A survey of European laboratories indicated that use of the original primer had not compromised *HFE *genotyping [13].

Homozygotes for C282Y and C282Y/H63D compound heterozygotes are known to be predisposed to the development of HH. As the significance of H63D in the homozygous state is unclear, H63D testing is currently only clinically useful in individuals heterozygous for C282Y. This would seem to imply that, ideally, C282Y should be tested for initially and H63D analysis performed on C282Y heterozygotes only as a reflex test. However, when there is a family history of HH, both mutations should be tested for, even if the index case is homozygous for C282Y and especially if extensive screening has not been performed in the family. The reason is that, due to the high carrier frequency of H63D (25% in the general population) [5], it is possible for both C282Y and H63D to be present in the same family and therefore for a family member to be a compound heterozygote at risk of iron overload. Such scenarios would be missed by reflex testing. H63D testing is appropriate when carrier status is required due to family history or for a spouse of a homozygote or heterozygote. Where a method detects both mutations concurrently, there is an obligation to report the complete result.

With respect to testing for mutations/variants other than C282Y & H63D, there is little published evidence that warrants such testing for diagnostic purposes. If a method picks up S65C, a lab may have an obligation to report the presence of this variant if it occurs in a compound heterozygote state with C282Y [14,15,18]. However, in the absence of any clinical merit, it would be best to avoid detecting S65C and hence the obligation to report on it is removed.

#### Reporting

Each laboratory has its own reporting format and guidance is available [19,20]. The following represents some basic guidelines for reporting HH genotypes based on reason for referral and resulting genotype. Points regarded as essential are highlighted in italics.

##### Diagnostic referral (i.e. affected individual) and genotype C282Y homozygous

*Reports should state, at a minimum, that this genotype is consistent with a diagnosis of HH*. Additional comments may refer to implications of the result for other family members, offer carrier testing and suggest that genetic counselling be considered. It is not considered appropriate to state that all relatives must/should be tested.

##### Diagnostic referral (i.e. affected individual) and genotype C282Y/H63D compound heterozygous

Some patients with this genotype have iron overload but to a lesser degree than C282Y homozygotes [7]. This genotype should therefore have a less weighted interpretation than for a C282Y homozygote referred on the same basis. Approximately 5% of patients with HH have this genotype but so also does 2% of the general UK population. Therefore, *the genotype is consistent with the presence of iron overload and may be diagnostic of HH once all other reasons for iron overload have been excluded (e.g. alcohol consumption, hepatitis C, hyperferritinaemia)*. Additional comments may refer to implications of the result for other family members, offer carrier testing and suggest that genetic counselling be considered. It is not considered appropriate to state that all relatives must/should be tested.

##### Diagnostic referral (i.e. affected individual) and genotype C282Y heterozygous

*The individual is a carrier of the C282Y mutation. This genotype makes a diagnosis of HH unlikely and such a diagnosis can only be made on a clinical basis*. It has been documented that about 25% of C282Y heterozygotes may exhibit mild to moderately raised indices of iron overload [5]. However, complications in C282Y heterozygotes due to iron overload are rare and may be influenced by additional factors, both genetic and environmental. In addition, other forms of iron overload and other types of haemochromatosis exist, therefore, a referral to a specialist unit may be suggested. Additional comments may refer to implications of the result for other family members and suggest that genetic counselling be considered. It is not considered appropriate to state that all relatives must/should be tested. The report should not state that a diagnosis of haemochromatosis is excluded.

##### Diagnostic referral (i.e. affected individual) and genotype H63D homozygous

This genotype is present in about 2% of the population and its significance remains uncertain. It has been suggested that H63D homozygotes have a slight risk of iron overload [16-18] and therefore *a diagnosis of haemochromatosis cannot be excluded and must be made on a clinical basis*. Additional comments may offer carrier testing for other family members and suggest that genetic counselling be considered. It is not considered appropriate to state that all relatives must/should be tested.

##### Diagnostic referral (i.e. affected individual) and genotype H63D heterozygous

*The individual is a carrier of the H63D mutation*. *This genotype makes a diagnosis of HH very unlikely and such a diagnosis can only be made on a clinical basis*. Other forms of iron overload and other types of haemochromatosis exist, therefore, a referral to a specialist unit may be suggested. Additional comments may refer to implications of the result for other family members and suggest that genetic counselling be considered. It is not considered appropriate to state that all relatives must/should be tested.*The report should not state that a diagnosis of haemochromatosis is excluded*.

##### Diagnostic referral (i.e. affected individual) and a normal genotype

*This genotype makes a diagnosis of HH very unlikely and such a diagnosis can only be made on a clinical basis*. Other forms of iron overload and other types of haemochromatosis exist therefore a referral to a specialist unit may be suggested.

##### Predictive referral (i.e. individual currently unaffected) and genotype C282Y homozygous

*The individual is at risk of developing iron overload*/*HH and it is recommended that the indices of iron overload (fasting, serum transferrin saturation and ferritin) be regularly monitored*. Suggested frequency for biochemical monitoring of individuals with this genotype is yearly [5]. Additional comments may suggest a referral to a specialist. The report may refer to implications of the result for other family members and suggest that genetic counselling be considered. It is not accurate to state that the individual has HH or will develop HH. It is not considered appropriate to state that all relatives must/should be tested.

Reported penetrance values (in terms of iron accumulation) for C282Y homozygosity range from 50% to 96% depending on the definition of iron overload used in the studies [6,7]. Since penetrance is age related and gender influenced, these factors must be incorporated into any genotype/phenotype correlation. Lyon and Frank [7] have summarised that the penetrance of C282Y homozygosity in males over 40 years is 95% for iron overload, when corrected for age and gender. For males under 40 years, the value is 80% with additional symptoms present in 12% of males in this age group. In the over 40 year female age group, 80% of C282Y homozygotes have iron overload with 13% exhibiting other symptoms. Iron overload is present in 39% of females under 40 years with no additional symptoms manifesting. In all cases, the penetrance of clinical haemochromatosis is much lower than the penetrance of iron overload [5-7].

##### Predictive referral (i.e. individual currently unaffected) and C282Y/H63D compound heterozygous

Five percent of individuals with HH have this genotype but so do 2% of the general population and some individuals with this genotype have iron overload but to a lesser degree than C282Y homozygotes [4-6]. *The individual may be at risk of developing iron overload/HH and it is recommended that the indices of iron overload (transferrin saturation, ferritin) be regularly monitored*. Suggested frequency for biochemical monitoring of individuals with this genotype is every three years [5]. Additional comments may suggest a referral to a specialist. The report may refer to implications of the result for other family members and suggest that genetic counselling be considered. It is not accurate to state that the individual has HH or will develop HH.

##### Predictive referral (i.e. individual currently unaffected) and C282Y heterozygous

*The individual is a carrier of the C282Y mutation. The individual is at less than the population risk of developing HFE related HH*. The presence of additional risk factors cannot be excluded. Suggested frequency for biochemical monitoring of individuals with this genotype is every five years [5]. The report may refer to implications of the result for other family members and suggest that genetic counselling be considered.

##### Predictive referral (i.e. individual currently unaffected) and H63D homozygous

It has been suggested that H63D homozygotes have a slight risk of iron overload, therefore, regular monitoring of the biochemical indices of iron overload (ferritin and transferrin saturation levels) may be suggested. The report may suggest that genetic counselling be considered. It is not accurate to state that the individual has HH or will develop HH.

##### Predictive referral (i.e. individual currently unaffected) and H63D heterozygous

*The individual is a carrier of the H63D mutation. The individual is at no increased risk of iron overload*. The report may refer to implications of the result for other family members and may suggest that genetic counselling be considered.

##### Predictive referral (i.e. individual currently unaffected) and normal genotype

*The individual is unlikely to develop HH*.

##### Carrier status referral and C282Y heterozygous

*The individual is a carrier of the C282Y mutation*. The report may refer to implications of the result for other family members and suggest that genetic counselling be considered. The report may suggest carrier testing for any partner.

##### Carrier status referral and H63D heterozygous

*The individual is a carrier of the H63D mutation*. The report may suggest carrier testing for any partner.

##### Carrier status referral and normal genotype

*The individual is not a carrier of the C282Y or H63D mutations*.

##### Information points on reports

Reports should contain information on the method used to generate the result, the sensitivity and specificity of the method if known and figures relating to haemochromatosis in the population being reported on, complete with references. These information points are best kept peripheral and not in the main body of the report.

#### Other issues

##### Testing of minors

Guidelines from The Clinical Genetics Society [21] and the American College of Medical Genetics [22] recommend not to test minors (under 16 years of age) for carrier status for late onset disorders. It is possible to offer to test parents and from this information, determine the approximate risk to each child of inheriting a genotype predisposing to the development of HH. The child may then choose to have their own carrier status investigated when they reach 16 years.

## Discussion/conclusion

An agreed set of best practice guidelines has now been developed for diagnostic, predictive and carrier testing for hereditary haemochromatosis and for reporting the results of such testing. The consensus and consultation processes worked well. From the feedback received following the workshop, remaining issues concern the real need to test for H63D and the carrier testing of spouses.

### H63D testing

The issue here is a mixture of clinicians who think that seeing families where only H63D is segregating may not be the most appropriate use of health care resources. Other specialties such as hepatology feel that the risk of iron overload in H63D homozygotes is evident in their clinical experience. The authors feel that the concerns over testing for H63D seem to be due primarily to clinical and laboratory workload implications. Some laboratories have adopted a reflex testing strategy while others have advocated use of streamlining referrals and testing for both mutations rather than reflex testing. As indicated in the agreed guidelines, when there is a family history of HH, both mutations should be tested for, even if the index case is homozygous for C282Y and especially if extensive screening has not been performed in the family. The reason is that, due to the high carrier frequency of H63D (25% in the general population) [5], it is possible for both C282Y and H63D to be present in the same family and therefore for a family member to be a compound heterozygote at risk of iron overload. Therefore, while reflex testing is not discouraged, testing for H63D only on patients heterozygous for C282Y, may be too constrained a policy. This implies that the benefit of reflex testing may be diminished if other considerations have to be taken into account (e.g. family history). It then remains a local decision as to whether to abandon reflex testing and test for both HFE mutations.

As the debate continues on the role (if any) of H63D in iron overload (there are individuals homozygous for this mutation who are iron overloaded but this may or may not be due to H63D), it is the view of the authors that we should continue to test for it. As the contribution of other genes to iron overload becomes established, it is envisaged that there will eventually be a call for an "iron overload" genetic screen as opposed to an HFE genetic screen.

It would be useful, if hepatologists and other disciplines published their clinical findings regarding H63D homozygotes. There may be a need to investigate other genes to ensure that any positive association is due to H63D alone.

### Testing of spouses

The value of a couple knowing that their child is at risk of a C282Y homozygous genotype was queried since there are no clinical consequences until well into adulthood and such testing only adds to laboratory workload and costs. The authors feel that many C282Y homozygotes and heterozygotes identified by HH testing will have adult offspring, so there could be some value in testing spouses to assess risks in such cases. If their spouse is shown not to carry either HFE mutation, offspring may be shown not to be at risk and therefore the need for, and cost of testing, is avoided.

## Abbreviations

ARMS, amplification refractory mutation system; EMQN, European Molecular Genetics Quality Network; HH, hereditary haemochromatosis; PCR, polymerase chain reaction; SSCP, single strand conformational polymorphism; UKNEQAS, United Kingdom National External Quality Assessment Service.

## Competing interests

The author(s) declare that they have no competing interests.

## Authors' contributions

CK designed the questionnaire, organised the meeting, drafted the guidelines, coordinated feedback on the guidelines and drafted the manuscript. DEB chaired the best practice workshop at the meeting. Both authors read and approved the final manuscript.

## Pre-publication history

The pre-publication history for this paper can be accessed here:


